# Drawing Direction Effect on a Task’s Performance Characteristics among People with Essential Tremor

**DOI:** 10.3390/s21175814

**Published:** 2021-08-29

**Authors:** Navit Roth, Orit Braun-Benyamin, Sara Rosenblum

**Affiliations:** 1 The Laboratory of Complex Human Activity and Participation (CHAP), Department of Occupational Therapy, University of Haifa, Haifa 3498838, Israel; rosens@research.haifa.ac.il; 2 Department of Mechanical Engineering, ORT Braude Academic College of Engineering, Karmiel 2161002, Israel; bborit@braude.ac.il

**Keywords:** essential tremor, drawing, lines, spiral

## Abstract

Essential tremor (ET) is a common movement disorder affecting the performance of various daily tasks, including drawing. While spiral-drawing task characteristics have been described among patients with ET, research about the significance of the drawing direction of both spiral and lines tasks on the performance process is scarce. This study mapped inter-group differences between people with ET and controls related to drawing directions and the intra-effect of the drawing directions on the tremor level among people with ET. Twenty participants with ET and eighteen without ET drew spirals and vertical and horizontal lines on a digitizer with an inking pen. Time-based outcome measures were gathered to address the effect of the drawing directions on tremor by analyzing various spiral sections and comparing vertical and horizontal lines. Significant group differences were found in deviation of the spiral radius from a filtered radius curve and in deviation of the distance curve from a filtered curve for both line types. Significant differences were found between defined horizontal and vertical spiral sections within each group and between both line types within the ET group. A significant correlation was found between spiral and vertical line deviations from filtered curve outcome measures. Achieving objective measures about the significance of drawing directions on actual performance may support the clinical evaluation of people with ET toward developing future intervention methods for improving their functional abilities.

## 1. Introduction

Essential tremor (ET) is one of the most common movement disorders, with a prevalence of about 5% at the age of 65 and above [1]. ET mainly causes posture and kinetic tremor of 4–12 Hz [2,3] in the upper limbs, but other body parts may be also affected [4,5]. In addition to the social implications, tremor may interfere with the ability to perform various activities of daily living (ADL) [4,6,7]. Louis and colleagues used the disability questionnaire, which includes varied ADL tasks, and found that above 70% of participants with ET reported disability in at least one task [6], while 68% of them reported difficulty or modification or change in efficiency in writing [6]. Bain and colleagues also found that a high percentage of people (−85%) reported difficulty in writing [4]. Drawing is another grapho-motoric task that requires hand movements while holding the pen against the paper/surface. Although drawing tasks are not included in some disability questionnaires (e.g., the Columbia University Assessment of Disability in Essential Tremor )CADET)) disability questionnaire [8]), they may be necessary as daily activities. Examples are sketching a graphic description or even underlining a sentence on paper.

Spiral- and straight-line-drawing features of people with ET may support the clinical evaluation process [9]. Indeed, several severity assessment scales, such as the Tremor Research Group Essential Tremor Rating Assessment Scale (TETRAS) and the Bain and Findley spirography scale, include visual assessment of a spiral task [10,11,12]. In recent years, digital analysis of drawing and writing tasks has been implemented to improve accuracy and obtain more objective quantitative measures of performance related to tremor. These methods include digital analysis of scanned spirals, without a time reference [13,14] or using digital graphic boards or even an iPad, that enables analyzing the sampled data in time [15,16]. Digital graphic boards enable data collection of pen tip location, pressure, and tilt angles [17]. Various data analysis techniques have been used, including both Cartesian (*y*- vs. *x*-position) and polar views (radius vs. angle) of pen tip location coordinates [13,15,18,19,20,21,22,23,24,25]. Several spiral frequency and time-based outcome measures have been reported, including change in the radius per angle, radial error, and deviation analysis from an optimal or pre-drawn spiral. Starting point center deviation has also been addressed [13,25]. Variability in the spiral width [26] and spiral main tremor axis [27,28] have also been investigated.

Research has been conducted to validate whether visual spiral scores and digital spiral analysis can support the clinical diagnostic process. Spiral features from digital assessments, such as peak amplitude from velocity spectral curves and residual deviation and fluctuation characteristics from radial fitted curves, have been found to correlate with visual rating scores when applying logarithmic relations [19,25]. Spiral scores have also been used by researchers to evaluate changes in tremor severity over time and showed that spiral scores increase [29]. Nevertheless, it seems that little data have been published comparing digital spiral outcome measures between ET and control groups and in comparison to other drawing tasks, such as lines.

Although drawing straight lines with a pen or a digital instrument is a more common daily task, these tasks (with or without tracing) have been evaluated less often in previous studies and tremor severity scales compared to the spiral task.

Other versions of line trajectory drawing have been used, such as drawing a line from one point to another [16,20]. Ulmanova and colleagues [16] included a task of drawing a line between two dots and found that the tremor magnitude is sensitive when comparing ET and control groups. Another study showed that velocity in the horizontal and vertical lines is lower in the ET group compared to the control group [20]. Results comparing drawing tasks may shed light on whether characteristics of other more common drawing tasks, such as straight lines, may contribute to the process of tremor assessment.

Drawing tasks involve both fine and gross motor skills and may include different hand postures, such as with or without the forearm leaning on the surface. They also have multiple directional features (e.g., spirals) or require a more distinct drawing direction (e.g., straight lines). In writing, the vertical direction is controlled by finger joints and the horizontal direction by the wrist [30,31,32,33]. Tremor in ET may manifest differently across joints of the upper limbs and in various directions of movement in each joint, for example, more flexion-extension than supination-pronation postural tremor in the wrist [34]. In addition, the tremor may affect grasping forces and prehension kinematics in precision grips [35]. Results from previous studies have shown a main tremor axis in spiral drawing [27,28] and a connection between the tremor axis and joint movements [36].

We did not find any published detailed correlation analyses between spiral and line outcome measures or the effect of the drawing direction (e.g., vertical vs. horizontal) on the actual performance in ET. Thus, the current study analyzes the drawing direction as a factor that may influence the performance for both spiral- and line-drawing tasks. Results may help improve understanding of how tremor affects various grapho-motoric tasks, as writing tasks are also controlled by vertical and horizontal movements (height, width, and horizontal movement on the paper), and whether there is an optimal or a preferred drawing or stroke writing direction that may help decrease the effect of the tremor on functional ability. For example, drawing and writing directions may change by changing the paper angle relative to the hand; thus, the therapist’s or the patient’s knowledge about a specific directional tremor in drawing tasks may help find a more optimal way to perform the task.

Thus, the present study’s aims were to (1) map differences in spiral and line characteristics between control and ET participants, (2) map the effect of drawing directions on the main outcome measures in spiral and line tasks, and (3) analyze relations between outcome measures of these tasks among participants with ET.

## 2. Methods

### 2.1. Participants

A total of 38 participants were included in the research, 20 in the ET group and 18 in the control group. Inclusion criteria for the ET group included age 20 years or older, with a physician’s diagnosis of ET and no other condition that could cause tremor, based on their reports. Control group inclusion criteria were age 20 years or older, without any known condition that could affect the upper limbs, including tremor, based on their reports.

Participants were recruited by advertising to the general population after receiving approval from the University of Haifa ethics committee, and they signed an informed consent form.

### 2.2. Instruments

**Figure 1:**
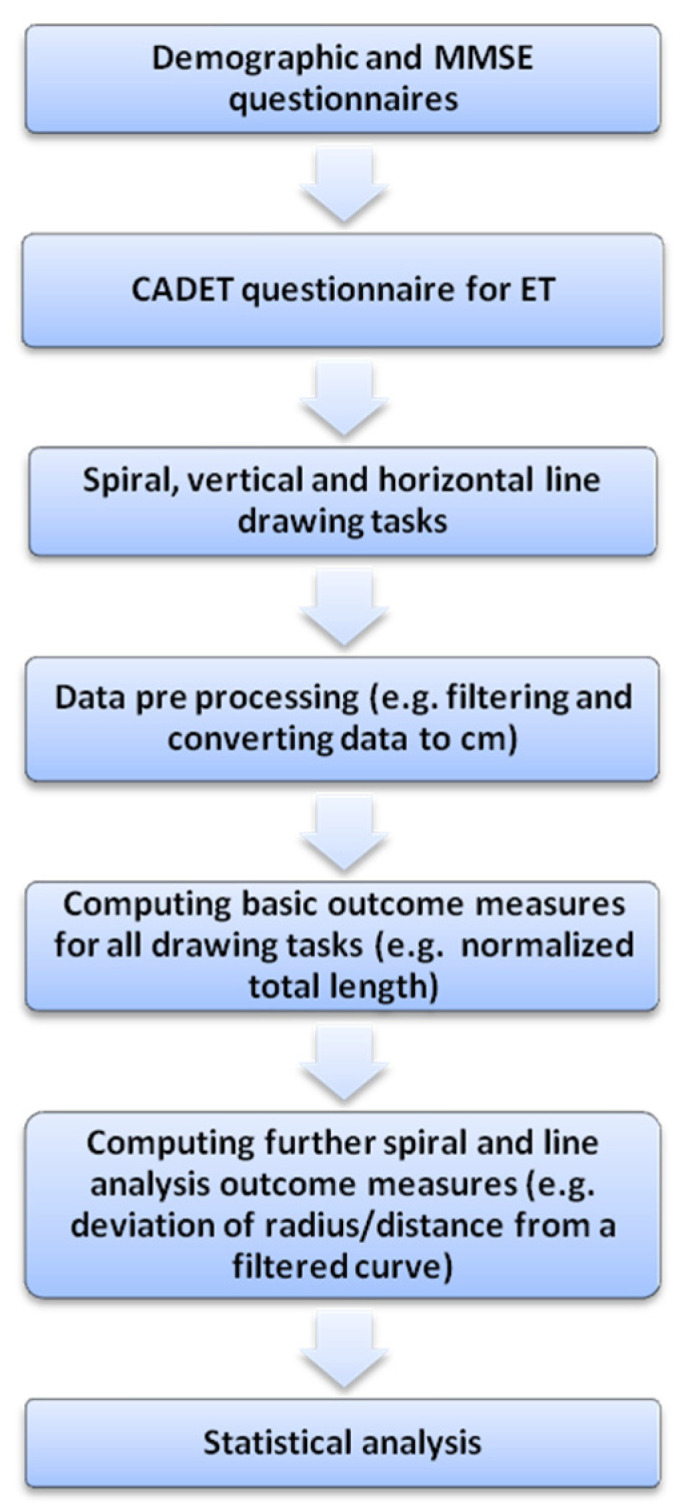
A flowchart describing research methods.

Participants who met the inclusion criteria completed a demographic and general information questionnaire, completed the Mini-Mental State Examination (MMSE) [37], and performed drawing tasks on a digital board. Participants with ET also completed the Columbia University Assessment of Disability in Essential Tremor )CADET) disability questionnaire [8]. A description of the methods is presented in the flowchart in Figure 1.

### 2.3. CADET Questionnaire

The validated CADET questionnaire was administered after approval by the corresponding author [8]. The 31-item questionnaire includes signing one’s name and writing, as well as other ADL tasks. Each item is scored from 0 to 2, with a maximum score of 100% (normalized by the maximum score of applicable items) [8]. Participants were also asked to list up to five ADL tasks that bother them the most due to tremor.

### 2.4. Drawing Tasks

Drawing tasks were executed on A4 paper sheets that were placed on an Intous2 digital board (Model XD-0912U) with a matching ink pen (WACOM, Kazo, Saitama, Japan) that was attached to a laptop. Data were captured from the graphic digital board at approximately 100 Hz via Computerized Penmanship Evaluation Tool (COMPET) handwriting performance analysis software [17,38]. Raw data from COMPET software included *x*- and *y*-coordinates (relative to board coordinates), the pen pressure level, and azimuth and tilt angles of the pen relative to the board. The raw data were further processed using MATLAB software and yielded outcome measures detailed below.

Participants were requested to perform the following drawing tasks while sitting in front of the table and using the hand they normally perform such tasks with during everyday life:

1. Tracking vertical and horizontal lines (3 cm and 3.5 cm long, respectively).
2. Drawing a spiral between pre-drawn spiral lines [19] with an inter loop width of approximately 1.5 cm [19] and an outer maximum radius of about 4.5 cm [18].

### 2.5. Data Processing of Drawing Tasks

Coordinates of the *x*- and *y*-directions in the drawing tasks were converted to centimeters using data from calibration pretests. For these tasks, a fourth-order Butterworth low-pass filter [20,39,40] with a cut-off frequency of 20 Hz was further applied. Four basic outcome measures were computed for all drawing tasks: (1) total task duration (on ground, continuous drawing on paper, after exclusion of data points); (2) total drawing length, computed by summing all distance intervals (between two sampling points); (3) normalized total length of the drawn curve, computed by normalizing the total drawing length by the distance between the first and last points (shortest distance; lengths were normalized due to the differences in the curve/line length created by participants, as not all of them started or ended at the exact same point); and (4) mean drawing velocity.

Some subject data were excluded in cases of multiple detachments from the board during drawing tasks. Partial subject data were excluded for the partial last spiral loop because of detachments and for the participants who drew one additional loop. These yielded *n* = 19 for the line tasks and *n* = 18 for the spiral tasks in the ET group.

As we wanted to compare the ET and control groups and address different tasks while focusing on the functional perspective, we defined additional outcome measures for each task, analyzing the created drawings. Some of these outcome measures are based on previous work, as detailed below.

#### 2.5.1. Spiral Task: Data Processing and Calculations

Spiral task analysis included the use of a polar view: radius (*r*) and angle (*θ*). The spiral radius was calculated in reference to the first point (*x*_0_, *y*_0_). As the task did not include a defined starting point, most participants started the spiral (first point) a few millimeters next to the center of the pre-drawn spiral. Thus, we compared all radius results with and without correcting for center deviation. The spiral angle was defined as the calculated angle between the radius and the horizontal axis of the board.

For the spiral-drawing task, four additional main outcome measures were computed:

**a. Spiral radial deviation from an estimated linear line.** This was examined as a measure of the deviation from a linear curve (describing the radius–angle connection of the predrawn spiral). An estimated line was created by a linear-fit estimation (*rl*) for the radial curvature (radius vs. angle). Spiral radial deviation was determined by calculating the normalized sum of square error (*SSE*) of the actual drawn spiral (*r*) and the estimated line (*SSE*_spl_ = Σ(*r* − *rl*)^2^). The *SSE* calculation included a radius of *r* > 0.4 cm. *SSE*_spl_ was then normalized by the number of task samples (*nSSE*_spl_).

**Figure 2:**
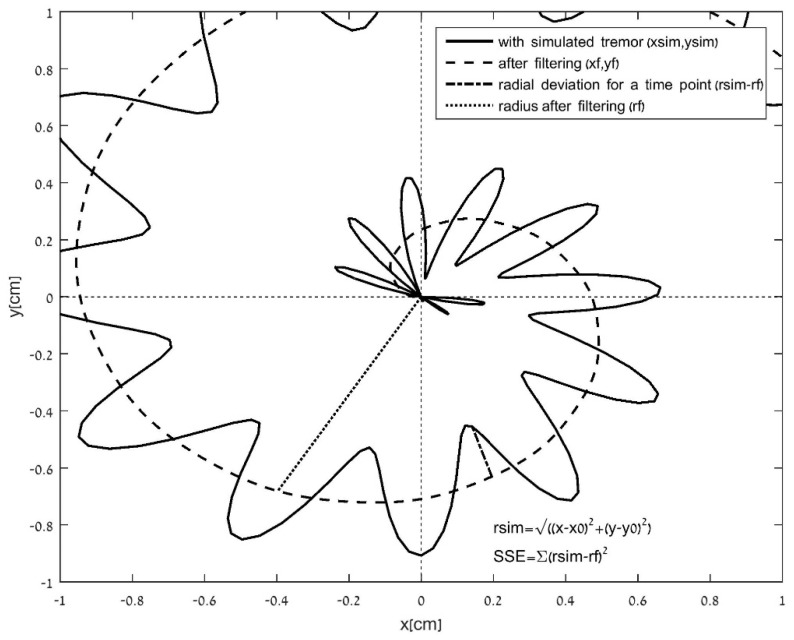
A 1 cm × 1 cm window of a simulated spiral, with an added radial tremor of 7 Hz and a 4 Hz cut-off filtered signal. Deviation of the spiral radius from the radius of the curve after filtering is computed as the normalized sum of square radial deviations of all points (spiral outcome measure b).

**b. Deviation of the spiral radius (distance curve) from a filtered curve.** This deviation was defined by the measure of the normalized SSE between the computed radius (*r*) and the filtered radius (*rf*) data (*SSE*_spf_ = Σ(*r* − *rf*)^2^). As in the first measurement, *SSE*_spf_ was normalized by the number of task samples (*nSSE*_spf_). The radius/distance was filtered in two ways: (b1) a low-pass filter with a 4 Hz cut-off frequency, as described in Figure 2, and (b2) a moving average fitted curve (with a window of 20 data points).

The mean-square deviation of the radial error and use of a low-pass filter and the moving average for a fitted curve (radius or velocity) have been addressed previously [14,25,41].

**Figure 3:**
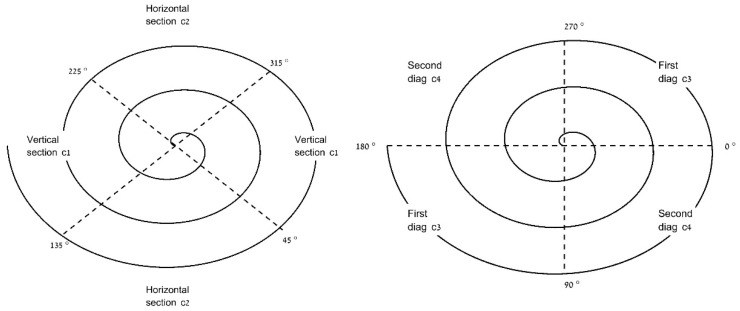
An illustrative example of the spiral sections. The angle is defined in the clockwise drawing direction: (**left**) vertical and horizontal sections c1 and c2; (**right**) diagonal sections c3 and c4. (c1) Vertical section: 315–345° and 135–225°. (c2) Horizontal section: 45–135° and 225–315°. (c3) First diagonal: 270–360° and 90–180°. (c4) Second diagonal: 180–270° and 0–90°.

**c. Deviation of the spiral radius from the filtered curve for vertical/horizontal diagonal sections.** The spiral was divided into horizontal and vertical sections and into diagonal sections to investigate the drawing direction effect and relate to the expected main spiral axis [27,28]. The normalized SSE was then computed, as described above, for each section. The sections were defined by circle angles, as presented in Figure 3: (c1) deviation of the spiral radius from the filtered curve in vertical sections of 315–45° and 135–225°; (c2) deviation of the spiral radius from the filtered curve in horizontal sections of 45–135° and 225–315°; (c3) deviation of the spiral radius from the filtered curve, first diagonal: 270–360° and 90–180°; and (c4) deviation of the spiral radius from the filtered curve, second diagonal: 180–270° and 0–90°.

The diagonal sections were analyzed by dividing according to left- and right-hand drawings.

**d. Average derivative of the spiral radius by angle.** The average derivative of the spiral radius by angle (d*r*/dθ) [23] was also calculated after converting to radians.

**e. The visual spiral score.** The score was computed by the average scores of two independent raters using the TETRAS scale spiral score [11] ranging from 0 for “normal” to 4 for “figure not recognizable.”

#### 2.5.2. Line Task: Data Processing and Calculations

For vertical and horizontal line tasks, two additional outcome measures were computed. These outcome measures were defined to address the tremor amplitude and the ability to draw a straight line from a functional perspective:

**a. Deviation from an estimated straight line as a measure of the ability to draw a straight line.** This was defined by the measure of the normalized *SSE* from an estimated/fitted linear line (*SSE*_ll_ = Σ(*e*)^2^). The errors were calculated from the difference between the actual *x*-value and the estimated *x*-value for the vertical lines (*e* = *x* − *ex*) and the actual *y*-value and the estimated *y*-value for the horizontal lines (*e* = *y* − *ey*). This was defined based on the thought that while drawing a vertical line (*x* = constant), the deviations in the horizontal direction are most unwanted and for the horizontal lines (*y* = constant), the vertical deviations are more relevant. As in the spiral tasks, *SSE*_ll_ was normalized by the number of task samples (*nSSE*_ll_).

**Figure 4:**
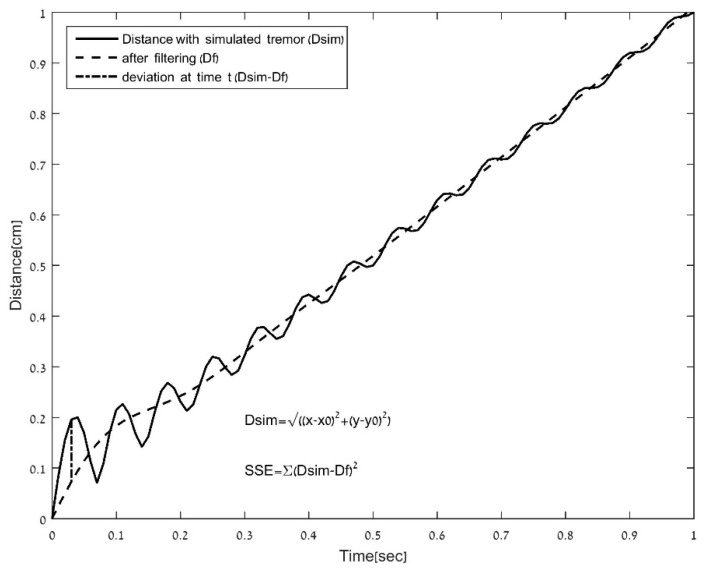
A 1 cm window of the distance (data point to the initial point) of a simulated vertical line with a tremor of 7 Hz and a 4 Hz cut-off filtered signal. Deviation of the distance curve from a curve after filtering was computed as the normalized sum of square deviations of all points (line outcome measure b).

**b. Deviation of the distance curve from a filtered distance curve.** The distance (D) was defined from each data point to the initial point (*x*_0_, *y*_0_). Deviation, as described in Figure 4, was computed for both line types by the measure of the normalized *SSE* between the distance curve and the filtered distance (*Df*) curve. The distance data were filtered, and the *SSE* (*SSE*_lf_ = Σ(*D* − *Df*)^2^) were normalized (*nSSE*_lf_) as in the spiral task: (b1) a low-pass filter of 4 Hz and (b2) a moving average fitted curve (with a window of 20 data points).

### 2.6. Statistical Analysis

Statistical analysis was carried out using SPSS software ver. 25 (IBM Corp., Armonk, NY, USA) and included descriptive and frequency analyses, Mann–Whitney tests for comparison between groups, and Wilcoxon tests for comparison within groups. The Spearman test was used to analyze the correlation between outcome measures of the ET group. A-Parametric tests for both groups used a sample size of ≤20, and most of the outcome measure results were not normally distributed.

## 3. Results

### 3.1. Demographics, MMSE, and CADET Questionnaire

Demographic data and CADET questionnaire scores for the ET group (*n* = 20) have been previously described (Roth and Rosenblum in process, [42]). Data for both groups are presented in Table 1.

In addition, 20% of the participants reported drawing or painting tasks as one of the five tasks that bother them the most due to tremor.

### 3.2. Drawing Tasks

#### 3.2.1. Spiral Analysis

While performing the spiral task, 10% of the participants with ET added support of the other hand on the drawing hand.

The number of ground strokes (continuous drawing on paper) for the ET group in the spiral task ranged from 1 to 3 (*n* = 18). The total task duration was 9.17 ± 4.00 s and 7.26 ± 2.53 s for the ET and control groups, respectively, while the total drawing length was 34.54 ± 5.90 cm (ET) and 33.08 ± 3.45 cm (control).

As shown in Table 2, the Mann–Whitney test yielded a significant difference for the average derivative of the spiral radius by angle and for deviation of the spiral radius from the filtered curve of the spiral radius, even when computed by the moving average (*p* < 0.05) and for all defined sections. No significant differences were found for the spiral radial deviation from an estimated linear line (even with starting point correction) or the normalized length of the drawn spiral. All these measures were higher among the ET group than in the control group. Comparing differences of the deviation of the spiral radius from the filtered curve between sections found a significant difference between horizontal and vertical spiral sections (Z = −3.72, *p* < 0.001 for ET and Z = −3.29, *p* = 0.001 for control). Although the mean of this outcome measure was higher in the first diagonal for right-hand drawing, the difference was not significant (Z = −1.7, *p* = 0.088 for ET).

**Figure 5:**
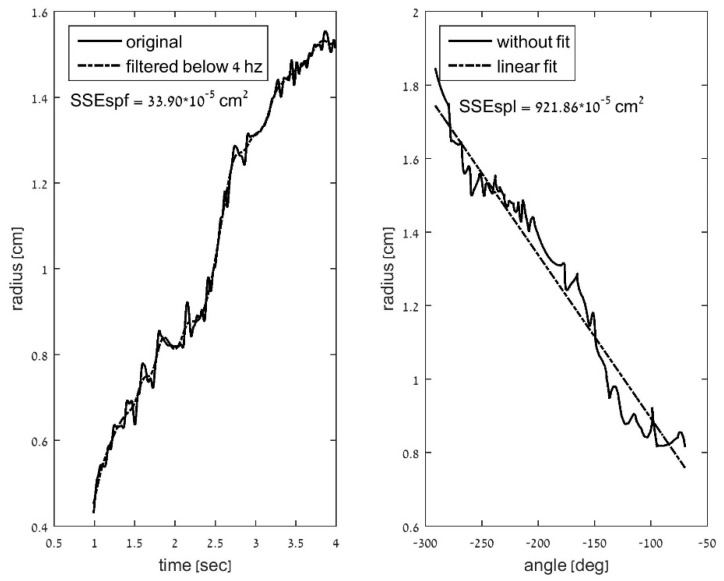
The 300 data points of radial curves for one participant from the ET group: computed radius (*r*) and filtered radius (*rf*) vs. time (**left**); computed radius (*r*) vs. angle and the linear-fit estimation (*rl*) (**right**).

An example for partial curves of the computed radius (*r*) and the filtered radius (*rf*) as well as the linear-fit estimation (*rl*) for one participant from the ET group are presented in Figure 5.

#### 3.2.2. Analysis of Lines

Results of the lines are detailed in Table 3, including inter-group and intra-task comparisons between vertical and horizontal tasks. While performing these tasks, 10% of ET participants used support of the other hand while drawing. In the vertical line task, the number of ground strokes were one in the ET group (*n* = 19) and ranged from one to two in the control group (*n* = 18). In the horizontal line, the range was one to three and one to two, respectively. The total task duration was 2.15 ± 0.75 s for vertical (V) lines and 2.51 ± 0.93 s for horizontal (H) lines for the ET group and 2.24 ± 0.70 s (V lines) and 2.68 ± 1.09 s (H lines) for the control group. The total drawing length was 3.62 ± 0.62 cm (V lines) and 4.18 ± 0.51 cm (H lines) for the ET group, whereas they were 3.27 ± 0.34 cm (V lines) and 3.86 ± 0.57 cm (H lines) for the control group.

As shown in Table 3, comparing data between groups, a significant difference was found for the normalized length for vertical lines and for deviation of the distance curve from a filtered curve for both vertical and horizontal lines and for the filtering method (*p* < 0.01 for the moving average). All these measures were higher among the ET group in comparison to the control group.

Comparing data within the ET group between horizontal and vertical lines, significant differences were found for the normalized total length of the drawn line, deviation from an estimated straight line, and deviation of the distance curve from the filtered curve (but not by using the moving average). A higher mean value was found for the deviation from an estimated straight line in the horizontal line but a higher deviation of the distance curve from the filtered curve for the vertical line.

### 3.3. Correlation Analysis between Spiral and Lines Task Measures for the ET Group

Correlation analysis was performed between the main outcome measures of spiral and line tasks (Table 2 and Table 3). Correlation results for *p* ≤ 0.05 are presented in Table 4.

As seen from Table 4, a significant medium-to-high correlation (*p* < 0.01) was found between spiral and vertical line deviations from the filtered curve outcome measure.

In addition, a significant medium-to-high correlation was found between the average derivative of the spiral radius by angle and the normalized total length of the vertical line (*p* < 0.01, *r* = 0.686), the normalized total length of the horizontal line (*p* < 0.01, *r* = 0.814), deviation of the distance curve from the filtered curve for the horizontal line (*p* < 0.05, *r* = 0.486), and deviation from an estimated straight line in the horizontal task (*p* < 0.01, *r* = 0.637). Addressing the visual rating, a significant medium-to-high correlation was found between the tremor severity visual score of the spiral drawing and deviation of the distance curve from the filtered curve for the vertical (*p* < 0.01, *r* = 0.715) and the horizontal (*p* < 0.01, *r* = 0.614) lines for the ET group. Correlation analysis between drawing task outcome measures and CADET questionnaire scores did not yield a significant result (the highest coefficient was *r* = 0.44, *p* = 0.07 for the normalized total length of the spiral).

## 4. Discussion

This study focused on analyzing drawing tasks that combine various directional joint movements and drawing directions. These tasks are part of our daily activities, and as ET becomes more prevalent after the age of 65, grapho-motor activities may become more relevant as part of leisure activity performance. Furthermore, smartphones and other digital instruments require touch-screen activities using either fingers or digital pens, which implement different grapho-motoric tasks, such as connecting points [43,44]. As line tasks involve short lines and to address functional outcome measures that describe how close a drawing is to an optimum estimated or desired curve, we defined time-based and not frequency-based outcome measures.

Although participants were requested to draw spirals between (and not on) the lines, in the spiral tasks, we explored the deviations from an estimated spiral by applying a linear fit in the radial view (radius vs. angle), as in previous studies. When we compared the drawn spiral in the radial view to the linear-fit estimation, a higher mean deviation was found for the control group but the results were not significant. In previous research regarding width variability in spiral drawing, a higher variability in the spiral width was found in ET participants compared to controls [26]; thus, we expected ET results to be higher. However, this defined deviation outcome measure captures both tremor fluctuations and general curve changes (closer and away from the predrawn spiral line). When we addressed and analyzed results with center (starting point) correction, deviation results decreased, as expected, but there was no effect on significance, and results were still higher for the control group. Deviation from an optimal linear curve when participants are requested to draw on the lines (and not between) may enable more clear conclusions on this outcome measure.

Analysis of the deviations from a filtered radial curve yielded a significantly higher mean for the ET group in all sections. In terms of section analysis, a significantly higher deviation was found in the horizontal sections compared to the vertical. In a study on tremor in multiple sclerosis, the authors included both squared and circular spirals and analyzed the velocities in different directions. They found tremor to be higher in the vertical and radial directions in the squared and circular spirals, respectively [41]. Addressing radial deviation in our current research, in the first and second diagonals (Figure 3), a significantly higher deviation was expected at the first diagonal for the right-hand-performed spirals, in accordance with the previous main axis detected at 2–3/1–2 o’clock for right-handed ET participants [27,28], but although the results were higher, there was no significance. This outcome measure addresses radial changes. Analyzing results from vertical/horizontal and diagonal sections, it seems that vertical movement is expressed more as radial changes in the horizontal section. The spiral task is a continuous drawing task with changing angles and requires or may involve different hand postures, joint movements, and directions, while the hand is not necessarily resting continuously on the paper. Thus, future analyses comparing the smaller and larger loops of the spiral may yield a wider scope of results.

In the line-drawing tasks, participants were requested to trace over the lines and draw short vertical and horizontal lines. The short vertical lines may require mostly fine motor movements of the finger joints while leaning on the wrist, but the horizontal lines may require more wrist movements. From the line results, deviation from an estimated linear curve was not found to be significant comparing between groups. Nevertheless, when analyzing results between lines in each group, values were significantly higher in the horizontal line for both groups. This outcome measure addresses vertical deviations from the horizontal lines and horizontal deviations from the vertical lines. Thus, in the vertical line, the tremor resulting from flexion-extension of the wrist and fingers is expressed less by deviation from the estimated line, i.e., less horizontal deviations with the vertical lines, and the vertical fluctuations in the vertical line are less obvious (as they are “more” on the line).

From the outcome measure of deviations from the filtered distance curve, results yielded a significant difference between groups. For both groups, a significantly higher deviation was found in the vertical line as a measure of the fluctuations in the distance curve. For the tremor effect, it seems that as a more flexion-extension tremor exists in the ET group, the deviations are more evident in the direction of movement for the vertical line. Accordingly, a significantly higher normalized distance was found for this line in the ET group, which indicates a longer curve created in this task. Comparing results between vertical and horizontal lines expresses how the tremor may manifest differently in different movements and drawing directions. This indicates that there may be a functional effect on the drawing direction that can affect each person differently. Thus, changing the performance characteristics when drawing lines, like changing the drawing direction (e.g., changing drawing angle/paper angle alignment), may optimize the desired outcome.

Comparing tremor characteristics to other tasks, such as tracking with a joystick, pouring, and posture, Bain and colleagues [45] showed that spiral scores correlate with tracking errors, water spilt, and tremor amplitude in posture tasks and that tremor characteristics of frequency and amplitude change between tasks. Others have found that frequency changes between writing, drawing, and posture tasks [24].

From correlation analysis of our results, vertical line deviation from the filtered distance correlated with spiral deviations from a filtered radial curve outcome measure. This seems to agree with the direction analysis described above for spirals and lines, as the tremor seems to be more vertical. Thus, these two outcomes are expected to correlate. Previous studies have validated spiral outcome measures against neurologists’ severity assessments [19,25]. We found a significant correlation between visual spiral scores and both vertical and horizontal line deviations from the filtered distance. Thus, as the vertical line task was shown to be sensitive between groups, we recommend that this task be validated against neurological tremor assessments in future research and be considered in drawing tasks for tremor evaluation, as they may be more familiar and prevalent in daily tasks.

We did not find significant correlations between drawing tasks outcomes and CADET questionnaire scores. As previous research found a correlation between hand acceleration measurements while drawing spirals and CADET questionnaire scores [8], further analysis is needed.

Our research had a few limitations. Participants were allowed to lean their hands on the table/board or to add support of the other hand on the drawing hand in all task types, which may influence performance characteristics between participants. Participants were also allowed to change the angle of the board to write on the paper in their normal manner. Thus, all direction analysis was in reference to the digital board axis and not to body orientation and could differ between participants. For the line tasks, lengths were short—about 3 cm. We recommend that future research include longer lines to investigate the effects of forearm posture and reaching movements. In addition, for drawing between lines in the spiral task, as aspects of performing the task, such as drawing freehand, between, or on lines, may influence the reliability of visual assessments in ET [46], it might influence results. Thus, we suggest future research including spiral on-line task vs. lines. Furthermore, our study included a limited number of participants, so we recommend conducting future studies with a larger sample.

Another issue in the context of future research is that in the present study, we used a graphic digital board for the measurements of drawing characteristics (pen tip), thus analyzing movements of the held object. Other measurement systems, such as accelerometers and gyroscopes [47,48], can provide additional biomechanical characteristics relating to the drawing hand. Thus, future research implementing these systems may provide a broad view by addressing the actual performance (drawing features) and drawing direction effect, as well as hand movement and tremor characteristics.

## 5. Conclusions

The present study aimed to broaden the view of the effect of the drawing direction on the actual performance of spiral and line tasks and investigate line tasks in comparison to validated spiral tasks. Our research supports previous digital analysis of spiral tasks by comparing ET and control groups. From the results, different drawing directions may result in varied characteristics of the actual performance, and it seems that a more vertical flexion-extension movement is manifested in both spiral and line tasks. This may help in better understanding and optimizing individual performance characteristics such as the drawing direction or hand posture. In addition, as vertical line results correlated with spiral outcome measures and were found sensitive when comparing groups, combining them with tremor severity assessments may be considered after further validation.

## Figures and Tables

**Table 1:** Participant demographics and CADET scores.

Characteristic	ET (*n* = 20)	Control (*n* = 18).
Sex, men	50%	38.90%
Right hand dominant ^a^	80%	83.30%
Age, mean ± SD (range)	64.9 ± 15.7 (23–81.7)	64.4 ± 10.9 (42.7–78.5)
Education, years, mean ± SD (range)	17.2 ± 3.4 (12–27)	18.0 ± 3.0 (12–24)
MMSE score, mean ± SD (range)	28.8 ± 1.4 (24–30)	29.1 ± 1.2 (27–30)
Years since noticing tremor (range)	21.9 ± 15.8 (4–55)	–
CADET disability questionnaire final score, mean ± SD (range)	32.4 ± 17.5% (5.8–66.7)	–

^a^ Dominant hand options: right, left, or both.

**Table 2:** Spiral characteristics (without first-point corrections).

	Characteristic/Outcome Measure	ET (*n* = 18)	Control (*n* = 18)	Comparison between Groups (MW)	
				Z	*p*
	Normalized length of the drawn spiral	8.59 ± 1.96	7.93 ± 1.04	−0.92	NS
	Mean drawing velocity ± SD (cm/s)	4.18 ± 1.17	5.21 ± 2.15	−1.30	NS
a	nSSEspl: spiral radial deviation from an estimated linear line (×10^−5^·cm^2^)	7218.64 ± 6258.27	9278.74 ± 9536.27	−0.38	NS
b1	nSSEspf: deviation of the spiral radius from the filtered curve (×10^−5^·cm^2^)	78.55 ± 184.30	21.02 ± 11.08	−3.04	0.002
c1	Deviation of the spiral radius from the filtered curve: vertical section (×10^−5^·cm^2^)	32.18 ± 22.88	18.10 ± 9.38	−2.66	0.008
c2	Deviation of the spiral radius from the filtered curve: horizontal section (×10^−5^·cm^2^)	114.73 ± 306.12	23.99 ± 13.89	−3.10	0.002
c3	Deviation of the spiral radius from the filtered curve: first diagonal for *rh*/*lh* ^a^ (×10^−5^·cm^2^)	*rh*-133.42 ± 374.32 (*n* = 15) *lh*-36.66 ± 13.69 (*n* = 3)	*rh*-23.02 ± 15.43 (*n* = 16) *lh*-18.58 ± 0.36 (*n* = 2)	−2.61 (*rh*)	0.009
c4	Deviation of the spiral radius from the filtered curve: second diagonal for *rh*/*lh* ^a^ (×10^−5^·cm^2^)	*rh*-42.2 ± 45.21 (*n* = 15) *lh*-42.50 ± 18.58 (*n* = 3)	*rh*-20.38 ± 10.15 (*n* = 16) *lh*-15.12 ± 3.74 (*n* = 2)	−2.49 (*rh*)	0.013
d	Average derivative of the spiral radius by angle (cm/rad)	2.46 ± 3.29	1.15 ± 1.20	−2.25	0.025
e	Tremor severity visual score of the spiral drawing	1.5 ± 1.03 (*n* = 20)	0.56 ± 0.45 (*n* = 18)	−3.25	0.001

^a^ *rh*/*lh*: right-hand/left-hand task performance.

**Table 3:** Vertical (V) and horizontal (H) line characteristics, Mann–Whitney sensitivity between groups (Z, *p*), and Wilcoxon analysis within group results for comparing vertical and horizontal line tasks.

	Outcome Measure	Task	ET (*n* = 19)	Control (*n* = 18)	Between-Group Comparisons (MW)		Within Group–between Line Comparison (W)	
					*p*	Z	Control	ET
	Normalized total length of the drawn line	V	1.35 ± 0.27	1.19 ± 0.13	0.033	−2.13	NS	Z = −2.05, *p* = 0.040
		H	1.21 ± 0.14	1.19 ± 0.14	NS	−0.76		
	Mean drawing velocity ± SD (cm/s)	V	1.99 ± 1.19	1.58 ± 0.43	NS	−0.94	NS	NS
		H	1.84 ± 0.61	1.59 ± 0.44	NS	−1.03		
a	nSSEll: deviation from an estimated straight line (×10^−5^·cm^2^)	V	83.52 ± 58.76	57.66 ± 44.34	NS	−1.42	Z = −2.03, *p* = 0.043	Z = −2.82, *p* = 0.005
		H	172.38 ± 117.75	112.25 ± 90.09	NS	−1.49		
b1	nSSElf: deviation of the distance curve from a filtered curve by filtering (×10^−5^·cm^2^)	V	173.70 ± 316.19	24.61 ± 16.34	0.001	−3.34	Z = −2.46, *p* = 0.014	Z = −2.29, *p* = 0.022
		H	43.87 ± 47.04	16.13 ± 5.65	<0.001	−3.71		

**Table 4:** Results of correlation analysis for the ET group between spiral, vertical (V), and horizontal (H) line tasks (*n* = 18).

	Outcome Measures		Spiral Task		
			Normalized Total Length of the Spiral	nSSEspf: Deviation of the Spiral Radius from the Filtered Curve	nSSEspl: Deviation from an Estimated Linear Line
Line tasks	Normalized total length of the line	V	N.S	0.575 *	NS
		H	0.525 *	NS	NS
	nSSElf: deviation of the distance curve from the filtered curve	V	N.S	0.668 **	NS
		H	N.S	0.536 *	NS
	nSSEll: deviation from an estimated straight line	V	N.S	N.S	NS
		H	N.S	N.S	NS

* *p* < 0.05, ** *p* < 0.01.

## Data Availability

The dataset analyzed in the current study is not publicly available due to the privacy of the participants.
